# Molecular characterization of CIMMYT maize inbred lines with genotyping-by-sequencing SNPs

**DOI:** 10.1007/s00122-016-2664-8

**Published:** 2016-02-05

**Authors:** Yongsheng Wu, Felix San Vicente, Kaijian Huang, Thanda Dhliwayo, Denise E. Costich, Kassa Semagn, Nair Sudha, Michael Olsen, Boddupalli M. Prasanna, Xuecai Zhang, Raman Babu

**Affiliations:** Maize Research Institute, Guangxi Academy of Agricultural Sciences (GXAAS), Nanning, 530007 Guangxi China; International Maize and Wheat Improvement Center (CIMMYT), Apdo. Postal 6-641, 06600 Mexico, DF Mexico; International Maize and Wheat Improvement Center (CIMMYT), P. O. Box 1041, Village Market, Nairobi, 00621 Kenya; CIMMYT-India, C/O ICRISAT, Patancheru, 502324 Andhra Pradesh India

## Abstract

*****Key message***:**

**Molecular characterization information on genetic diversity, population structure and genetic relationships provided by this research will help maize breeders to better understand how to utilize the current CML collection.**

**Abstract:**

CIMMYT maize inbred lines (CMLs) have been widely used all over the world and have contributed greatly to both tropical and temperate maize improvement. Genetic diversity and population structure of the current CML collection and of six temperate inbred lines were assessed and relationships among all lines were determined with genotyping-by-sequencing SNPs. Results indicated that: (1) wider genetic distance and low kinship coefficients among most pairs of lines reflected the uniqueness of most lines in the current CML collection; (2) the population structure and genetic divergence between the Temperate subgroup and Tropical subgroups were clear; three major environmental adaptation groups (Lowland Tropical, Subtropical/Mid-altitude and Highland Tropical subgroups) were clearly present in the current CML collection; (3) the genetic diversity of the three Tropical subgroups was similar and greater than that of the Temperate subgroup; the average genetic distance between the Temperate and Tropical subgroups was greater than among Tropical subgroups; and (4) heterotic patterns in each environmental adaptation group estimated using GBS SNPs were only partially consistent with patterns estimated based on combining ability tests and pedigree information. Combining current heterotic information based on combining ability tests and the genetic relationships inferred from molecular marker analyses may be the best strategy to define heterotic groups for future tropical maize improvement. Information resulting from this research will help breeders to better understand how to utilize all the CMLs to select parental lines, replace testers, assign heterotic groups and create a core set of breeding germplasm.

## Introduction

Molecular characterization of genetic diversity, population structure and genetic relationships among elite breeding materials within a given set of maize germplasm is useful for understanding how to use the assembled germplasm for further improvement, such as selecting parental lines, assigning heterotic groups and creating a core set of germplasm. Maize germplasm can be divided into two major groups—i.e., temperate and tropical germplasm—based on environmental characteristics, particularly day length. The International Maize and Wheat Improvement Center (CIMMYT) focuses mainly on tropical maize germplasm improvement. CIMMYT maize inbred lines (CMLs) are carefully selected for good general combining ability and a significant number of value-added traits such as drought tolerance, nitrogen use efficiency, resistance to disease and insect pests, and grain nutritional quality. Generally, two heterotic groups were classified within CMLs, i.e., the “A” group is dent kernel type and “B” group is flint kernel type. CIMMYT CMLs are widely used by public and private institutions worldwide, especially in developing countries (Ron Parra and Hallauer 1997; Warburton et al. 2002). From 1984 to 2003, CIMMYT developed and released 539 CMLs, which may well represent the total genetic diversity of improved tropical maize germplasm due to their wide distribution and great contribution to tropical maize breeding improvement.

Several molecular characterization studies of maize have been conducted using different germplasm collections and various kinds of low-to-medium density genotyping platforms (Lu et al. 2009; Mir et al. 2013; Semagn et al. 2012; Warburton et al. 2005, 2008; Wen et al. 2011, 2012; Wu et al. 2014; Xia et al. 2004, 2005; Yan et al. 2009). These studies have revealed the clear population structure among temperate and tropical/subtropical lines, the greater genetic diversity of tropical maize and the clear heterotic patterns in temperate maize germplasm. However, clear information on tropical maize germplasm is lacking for clustering patterns based on phenotype, environmental adaptation and heterotic group. Molecular marker analyses provide an important alternative approach for characterizing genetic diversity, population structure and genetic relationships among elite breeding materials within a given maize germplasm collection. However, most previous studies focused mainly on a specific temperate maize collection or on a global maize collection that did not include a sufficient number of CMLs to fully represent improved tropical maize. Comprehensive molecular characterizations of tropical maize germplasm collections which include a large number of CMLs or on a collection with all CMLs are essential.

Molecular characterization analyses can use various kinds of molecular markers, including restriction fragment length polymorphisms (RFLPs), amplified fragment length polymorphisms (AFLPs), simple sequence repeats (SSRs) or single nucleotide polymorphisms (SNPs) (Dillman et al. 1997; Reif et al. 2003; Warburton et al. 2002; Xia et al. 2004, 2005). More recently, chip-based SNP detection technology is being widely used in plant genetic applications including molecular characterization analyses, because of their low cost per data point, high genomic abundance, potential for high-throughput analysis and lower genotyping error rates. In maize, Illumina chip-based SNP detection platforms (i.e., GoldenGate containing 1536 SNPs and MaizeSNP50 BredChip containing 56,110 SNPs) have been used for molecular characterization analyses (Lu et al. 2009; Semagn et al. 2012; Wu et al. 2014). However, the GoldenGate Assay contains only 1536 SNPs and the maximum number of alleles at each locus is two, which may cause lower resolution in molecular characterization analyses. Molecular characterization with a large number of SNPs could overcome the deficiency of chip-based SNPs where the maximum number of alleles per locus is restricted to two. Furthermore, most current chip-based SNPs were developed based on the sequencing information of a set of temperate maize lines, which leads to ascertainment bias of the allele frequency and affects the resolution of genetic diversity and population structure analysis of tropical germplasm collections (Lu et al. 2009).

Genotyping-by-sequencing (GBS), a next-generation sequencing (NGS) technology, is a high-throughput, multiplex and short-read sequencing approach that reduces genome complexity via restriction enzymes and generates high-density genome-wide markers (~1 million) at a low per sample cost by tagging randomly shared DNA fragments from different samples with unique, short DNA sequences (barcodes) and pooling samples into a single sequencing channel (Elshire et al. 2011). Now used for generating large numbers of SNPs in many species, GBS has emerged as a powerful tool for different genetic applications, such as genetic diversity analysis, linkage mapping, association mapping and genomic selection (Crossa et al. 2013; Poland and Rife 2012; Romay et al. 2013; Zhang et al. 2015). An SNP calling pipeline called the Universal Network-Enabled Analysis Kit (UNEAK) was specially developed for species without a sequencing reference genome (Lu et al. 2013). Several studies have indicated that GBS SNPs called from the UNEAK pipeline provide great potential to exploit natural genetic diversity in species without a sequencing reference genome, such as switchgrass and yams (Lu et al. 2013; Girma et al. 2014). TASSEL-GBS Discovery/Production pipeline was specifically tailored to the GBS protocols to use reference genome sequencing information for SNP discovery and calling (Glaubitz et al. 2014). In maize, Romay et al. (2013) first investigated the genetic relationship of 2815 maize inbred accessions preserved at the USA national maize inbred seed bank by using a TASSEL-GBS SNP discovery pipeline with the temperate maize inbred, B73, as the reference genome.

One million SNPs on each DNA sample could be generated using GBS, which makes it possible to reduce ascertainment bias and improve the resolution of molecular characterization analyses in a tropical maize collection. In this study, 539 CMLs genotyped with GBS were used for molecular characterization analyses. The main objectives of this study were to: (1) understand the genetic diversity of a tropical maize collection comprising all CMLs; (2) investigate the population structure and heterotic patterns of this collection; (3) estimate the genetic relationships among the most important CMLs; and (4) assess how GBS could be utilized to perform genetic diversity analyses in tropical maize.

## Materials and methods

### Plant materials

A panel of 538 CMLs and 6 temperate inbred lines was used for molecular characterization analysis. CML250 was not included due to missing sample issues. Basic information on all of the lines including their environmental adaptation, number of lines in each geographic subset, grain color, grain texture and testers in each adaptation group is summarized in Table 1. Further information about all CMLs is available here: http://hdl.handle.net/11529/10246. All the released CMLs are publicly available to anyone in the world and could be requested through the CIMMYT Maize Germplasm Bank online seed ordering site: http://www.cimmyt.org/en/what-we-do/germplasm-and-seed/obtainseed.

**Table 1:** Environmental adaptation, geographic subset and phenotypic characterization information of 544 maize inbred lines

Environmental adaptation/ geographic subset	No. of lines	Grain color^a^		Grain texture^b^				Tester
		Y	W	D	SD	SF	F	
Mexico Lowland	241	92	149	68	51	29	93	CML161, CML165, CML247, CML254, CML287, CML413, CML416, CML419, CML420, CML421, CML422, CML423, CML451, CML491, CML494, CML495, CML503
Africa Lowland	22	8	14	6	0	0	16	
Asia Lowland	20	20	0	3	4	2	11	CML426, CML429, CML430, CML431, CML433
Mexico Subtropical	145	34	111	51	37	21	36	CML311, CML312, CML321, CML323, CML327, CML373, CML384
Africa Mid-altitude	58	0	58	9	22	17	11	CML202, CML206, CML395, CML444, CML522, CML537, CML539
South America	22	17	5	1	2	6	13	CML437, CML440
Highland Tropical	30	4	26	2	16	12	0	CML242, CML246, CML349
Temperate	6	6	0	3	0	0	3	B37, B73, B84, Mo17, C103, Oh43

^a^Grain color: *Y* = yellow and *W* = white

^b^Grain texture: *D* = dent, *SD* = semi-dent, *SF* = semi-flint and *F* = flint

Based on geographic information and environmental adaptation, 538 CMLs were classified into seven subsets: (1) Mexico Lowland: 241 lines developed in Mexico; (2) Africa Lowland: 22 lines developed in Kenya and Zimbabwe; (3) Asia Lowland: 20 lines developed in Thailand; (4) Mexico Subtropical: 145 lines developed in Mexico; (5) Africa Mid-altitude: 58 lines developed in Kenya and Zimbabwe; (6) South America: 22 lines developed in Colombia; and (7) Highland Tropical: 30 lines developed in Mexico. For convenience, we refer to the Mexico Lowland, Africa Lowland and Asia Lowland subsets as the Lowland Tropical subgroup; the Mexico Subtropical, Africa Mid-altitude and South America subsets as the Subtropical/Mid-altitude subgroup; and the Highland Tropical subset as the Highland Tropical subgroup, since they are from different CIMMYT maize breeding programs targeting similar environments. Six temperate maize inbred lines (i.e., B37, B73, B84, C103, Mo17 and Oh43) were referred to as the Temperate subgroup and used as a reference to assess the genetic divergence between temperate and tropical maize.

### SNP genotyping

For all the maize lines tested in this study, leaf samples bulked from 15 plants of each line were used for DNA extraction with a CTAB procedure (Tel-zur et al. 1999). DNA of all the samples was sent to the Cornell University Biotechnology Resource Center for GBS. A GBS protocol commonly used by the maize research community was applied in this study (Elshire et al. 2011). Genomic DNA was digested with the restriction enzyme *Ape*K1. GBS libraries were constructed in 96-plex and sequenced on Illumina HiSeq 2000. SNP calling was performed using TASSEL-GBS pipeline with B73 as the reference genome (Glaubitz et al. 2014) to generate a comprehensive genotype collection, the AllZeaGBSv2.7 Production Build (www.panzea.org). This collection includes genotypes of more than 60,000 maize samples. In this study, we focused on the subset of 539 genotyped CMLs, and un-imputed raw GBS data were used for further genetic characterization analyses. Initially, 955,690 SNPs evenly distributed on maize chromosomes were called for each line; 955,120 of them were assigned to chromosomes 1–10, and 570 of them could not be anchored to any of the 10 maize chromosomes. A filtered subset of 362,008 SNPs with minor allele frequency (MAF) greater than 0.05 was selected for genetic characterization analyses in this study. For all the maize lines tested in this study, un-imputed 955,690 SNP dataset and filtered 362,008 SNP dataset are publicly available on the CIMMYT Research Data Repository website: http://data.cimmyt.org/dvn.

### Data analysis

Allele frequency analysis of the 362,008 SNPs was carried out with TASSEL 5.0 software (Bradbury et al. 2007). The polymorphic information content (PIC) values of these 362,008 selected SNPs were calculated in Microsoft Excel 2010 according to the following formula to refer to the relative value of each marker with respect to the amount of polymorphism exhibited. This was described as:

1 $${\text{PIC}}_{I} = 1 - \mathop \sum \limits_{j = 1}^{n - 1} P_{ij}^{2} - \mathop \sum \limits_{j = 1}^{n - 1} \mathop \sum \limits_{k = j + 1}^{n} 2P_{ij}^{2} P_{ik}^{2} ,$$

where *P*_*ij*_ and *P*_*ik*_ are the frequencies of the *j*th and *k*th alleles of marker *i*, respectively; the summation extends over *n* alleles.

The pairwise relative kinship for all 538 CMLs was estimated with the entire data set of 362,008 SNPs using software TASSEL 5.0 (Bradbury et al. 2007) to reveal the genetic relatedness among all CMLs in the panel; Loiselle kinship (Yang et al. 2011) between each pair of lines was calculated. A relative kinship close to 0 indicates no relationship, and a value close to 1 indicates a closer relationship.

Heterozygosity rate and gene diversity for 362,008 selected SNPs were calculated using R Poppr package (Kamvar et al. 2014) to quantify the genetic variation of each tested maize line within each subgroup. The heterozygosity rate describes the proportion of heterozygous loci detected in each line, and gene diversity is defined as the probability that two alleles randomly chosen from the test samples are different.

Within the entire panel and each subgroup, the average linkage disequilibrium (LD) between SNPs on each chromosome was measured with 362,008 SNPs in TASSEL 5.0. Squared Pearson correlation coefficient (*r*^2^) between vectors of SNP alleles was used to assess the level of LD decay on each chromosome, and average LD decay distance across ten chromosomes with *r*^2^ = 0.1 was used to measure the difference of LD decay distance between the entire panel and other subgroups (Yan et al. 2009). A 50 kb slide window was used to determine the width of the window on one side of the start site, the spacing between two loci on the same chromosome was segmented in a distance of 50 kb and the average LD was assessed for each window (Bradbury et al. 2007).

Of the 362,008 filtered SNPs, 7497 were randomly selected, which were evenly distributed on ten maize chromosomes. An admixture model-based clustering method (Wang et al. 2008) using the software Structure V2.3.3 (Hubisz et al. 2009) with 7497 SNPs was applied for population structure analysis on all 538 CMLs for *K* number of clusters ranging from 1 to 7; each *K* was run three times with a burn-in period of 10,000 and 10,000 replications. The ad hoc statistic delta *K* (*ΔK*) was used to determine the number of clusters (Evanno et al. 2005). Population structure resulting from the admixture model-based clustering method was illustrated by software ClUMPP (Jakobsson and Rosenberg 2007) when *K* = 3 and lines with membership probabilities of more than 0.5 were assigned to the corresponding clusters (Wu et al. 2014).

Principal component analysis (PCA) based on 362,008 SNPs was performed with the R software (http://www.R-project.org) to assess the genetic relationship among all 544 maize inbred lines, and missing data in PCA were imputed to mean allele frequency. The first two principal components were illustrated for the entire panel and for each environmental adaptation.

The computation of Rogers’ genetic distance (Rogers 1972) between lines was performed with the 362,008 selected SNPs using R Poppr package (Kamvar et al. 2014). The genetic distance between the Lowland Tropical subgroup, Subtropical/Mid-altitude subgroup, Highland Tropical subgroup and Temperate subgroup was estimated based on the average genetic distance of all lines within each subgroup. The Unweighted Pair Group Method with Arithmetic Mean (UPGMA) trees for all 544 lines and selected important lines within each subgroup were constructed based on their Rogers’ genetic distances.

## Results

### SNP characteristics

The initial un-imputed GBS data included 955,690 SNPs for all maize inbred lines tested; 955,120 of them were evenly distributed on chromosomes 1–10, and the number of SNPs on each chromosome ranged from 148,752 on chromosome 1 to 67,126 on chromosome 10. The missing rate across genomes ranged from 57.61 % on chromosome 5 to 58.42 % on chromosome 10, with a mean value of 57.99 %, whereas the heterozygosity rate ranged from 0.41 % on chromosome 4 to 0.45 % on chromosomes 5, with a mean value of 0.43 % (Table 2). The remaining 570 SNPs that could not be mapped to any of the ten maize chromosomes were removed from the genetic characterization analyses performed in this study.

**Table 2:** Basic information on the datasets including 955,120 and 362,008 SNPs, respectively

Chromosome number	955,120 SNP dataset			362,008 SNP dataset		
	No. of SNPs	Missing rate (%)	Heterozygosity rate (%)	No. of SNPs	Missing rate (%)	Heterozygosity rate (%)
1	148,752	57.82	0.43	56,425	54.19	0.99
2	115,173	57.93	0.44	44,853	54.39	1.00
3	108,224	57.95	0.44	42,097	54.59	1.00
4	94,726	58.10	0.41	33,199	54.22	1.02
5	110,328	57.61	0.45	42,528	54.05	1.03
6	76,475	58.22	0.42	28,779	54.64	0.98
7	80,517	57.72	0.43	30,438	54.20	0.99
8	81,431	57.94	0.42	30,853	54.44	0.98
9	72,368	58.19	0.43	27,580	54.69	0.99
10	67,126	58.42	0.43	25,256	55.04	1.01
Total	955,120	–	–	362,008	–	–
Average	95,512	57.99	0.43	36,200.8	54.45	0.99

**Fig. 1:**
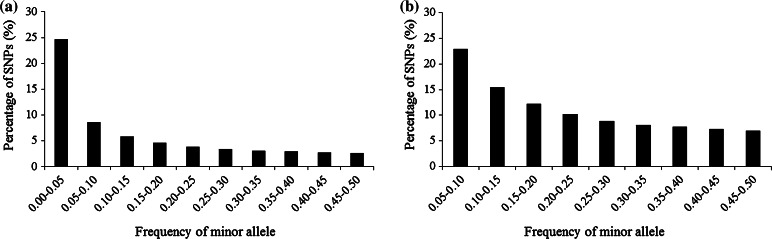
Frequency distribution of minor allele in 544 maize inbred lines based on: **a** 955,120 SNP dataset; **b** 362,008 SNP dataset

Of the 955,120 SNPs, 362,008 with an MAF greater than 0.05 were selected for further genetic characterization analyses. Only population structure analysis were conducted with 7497 evenly distributed SNPs, which were randomly selected from the 362,008 SNP dataset. Detailed information on a subset of 362,008 SNPs (i.e., number of SNPs on each chromosome, missing rate and heterozygosity rate) is provided in Table 2. Compared with the subset of 955,120 SNPs, the missing rates in the 362,008 SNP subset was a little lower. The heterozygosity rate in the 362,008 SNP dataset was a little higher, because non-polymorphic and complete missing SNPs were deleted. The heterozygosity rate in the 362,008 SNP subset was 0.99 %; this value is normal for a maize inbred line collection. MAF information for the 955,120 and 362,008 SNP datasets is shown in Fig. 1. GBS allows more than two alleles called per marker loci, since calls are always made on the same DNA strand. The number of alleles per marker loci ranged from 0 to 6 in the 955,120 SNP dataset; 597,213 SNPs were actually polymorphic (MAF greater than zero), 499,297 SNPs had an MAF above 0.01 and the average MAF was 0.09, with continuous distribution classes from 0.00 to 0.50 at intervals of 0.05. In the 362,008 SNP dataset, the average MAF was 0.22, with continuous distribution classes from 0.05 to 0.50 at intervals of 0.05, the number of alleles per marker loci ranged from two to six, 582,249 marker loci had more than two alleles, the proportion of marker loci having more than two alleles was 16 and 95 % of them were indels. The third (or more) alleles were removed with additional filtering for the other genetic diversity analysis. The PIC values varied from 0.09 to 0.38 in the 362,008 SNP dataset, with a mean value of 0.25.

### Relative kinship

**Fig. 2:**
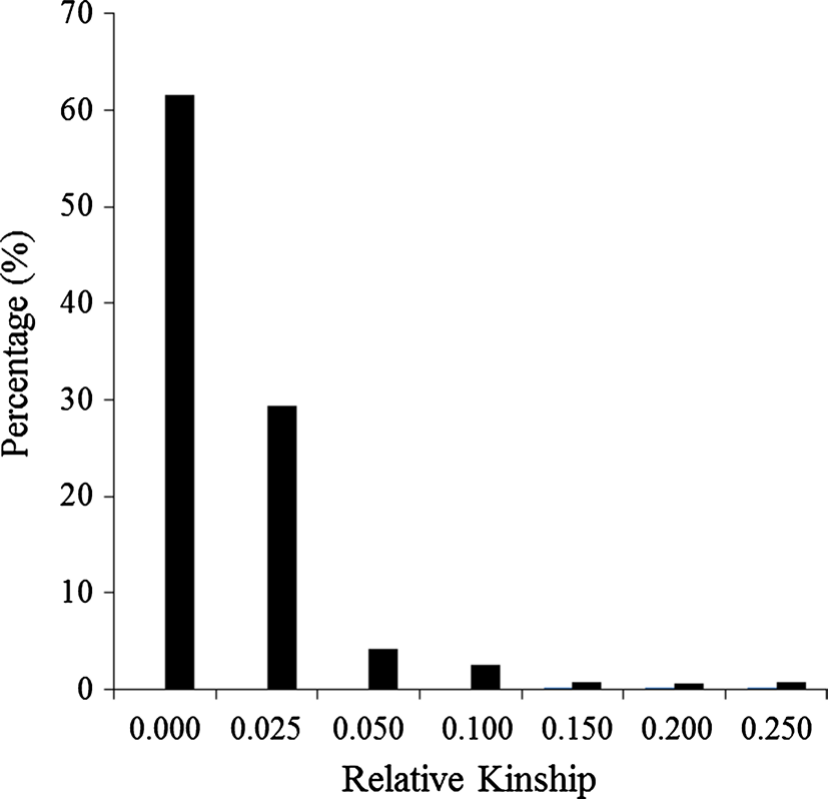
Distribution of pairwise relative kinship for 544 maize inbred lines calculated using 362,008 filtered SNPs

The distribution of pairwise relative kinship for 544 maize inbred lines estimated with 362,008 SNPs is shown in Fig. 2. Results showed that 62 % of the pairwise relative kinships were equal to 0; 29 % of them ranged between 0 and 0.025, 4 % ranged between 0.025 and 0.050 and only about 5 % were above 0.050. This information indicates that most of the lines in the entire panel are either not related or only distantly related to each other.

### LD decay

**Fig. 3:**
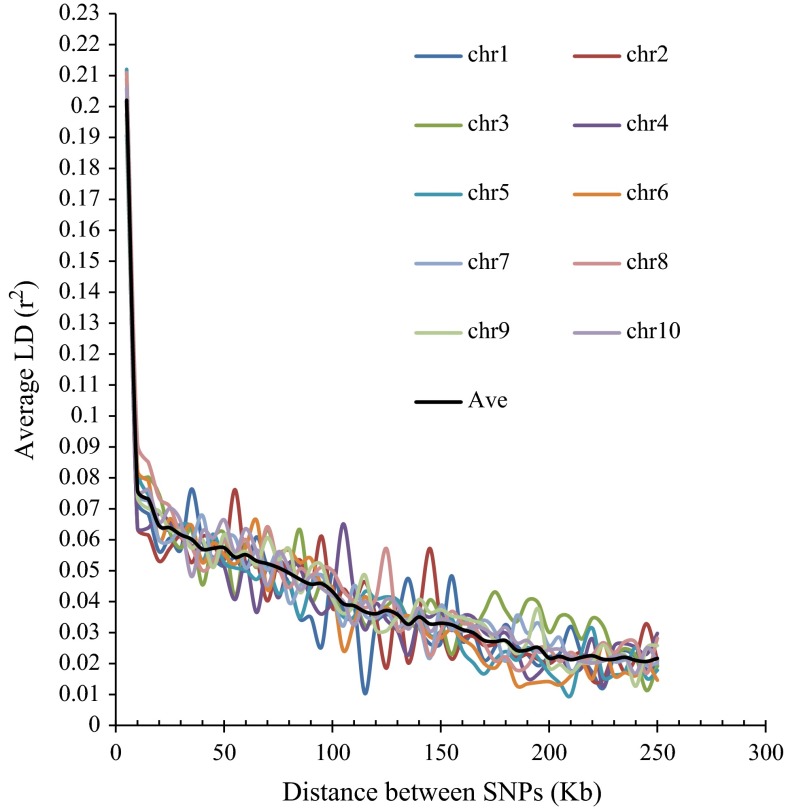
Whole-genome LD in the entire panel. LD within and over chromosomes is given in physical distance of 50 kb

LD for the entire panel and within chromosomes was measured (Fig. 3). LD decay varied across the ten chromosomes, as well as across different genetic regions within chromosomes. The average LD decay distance over all ten chromosomes in the entire panel with *r*^2^ = 0.1 was 3.76 kb. In Lowland Tropical subgroup, Subtropical/Mid-Altitude subgroup and Highland Tropical subgroup, the average LD decay distances over all ten chromosomes with *r*^2^ = 0.1 were 4.31, 3.12 and 3.56 kb, respectively (Table 3). The Lowland Tropical subgroup has the largest LD decay distance, whereas the Subtropical/Mid-altitude subgroup has the smallest LD decay distance. Due to limited number of samples in the Temperate subgroup, the LD decay distance of this subgroup was not estimated.

**Table 3:** Polymorphic information content (PIC), heterozygosity rate, gene diversity and average LD decay distance within each subgroup and the entire panel including all 544 lines

Group	No. of lines	PIC	Heterozygosity rate	Gene diversity	LD decay distance (kb)
Entire panel	544	0.25 ± 0.09	0.01 ± 0.01	0.31 ± 0.14	3.76
Lowland Tropical	283	0.24 ± 0.10	0.01 ± 0.02	0.30 ± 0.14	4.31
Subtropical/Mid-altitude	225	0.25 ± 0.10	0.01 ± 0.02	0.30 ± 0.14	3.12
Highland Tropical	30	0.21 ± 0.13	0.01 ± 0.03	0.26 ± 0.18	3.56
Temperate	6	0.17 ± 0.16	0.00 ± 0.04	0.21 ± 0.21	–

### Population structure analysis

**Fig. 4:**
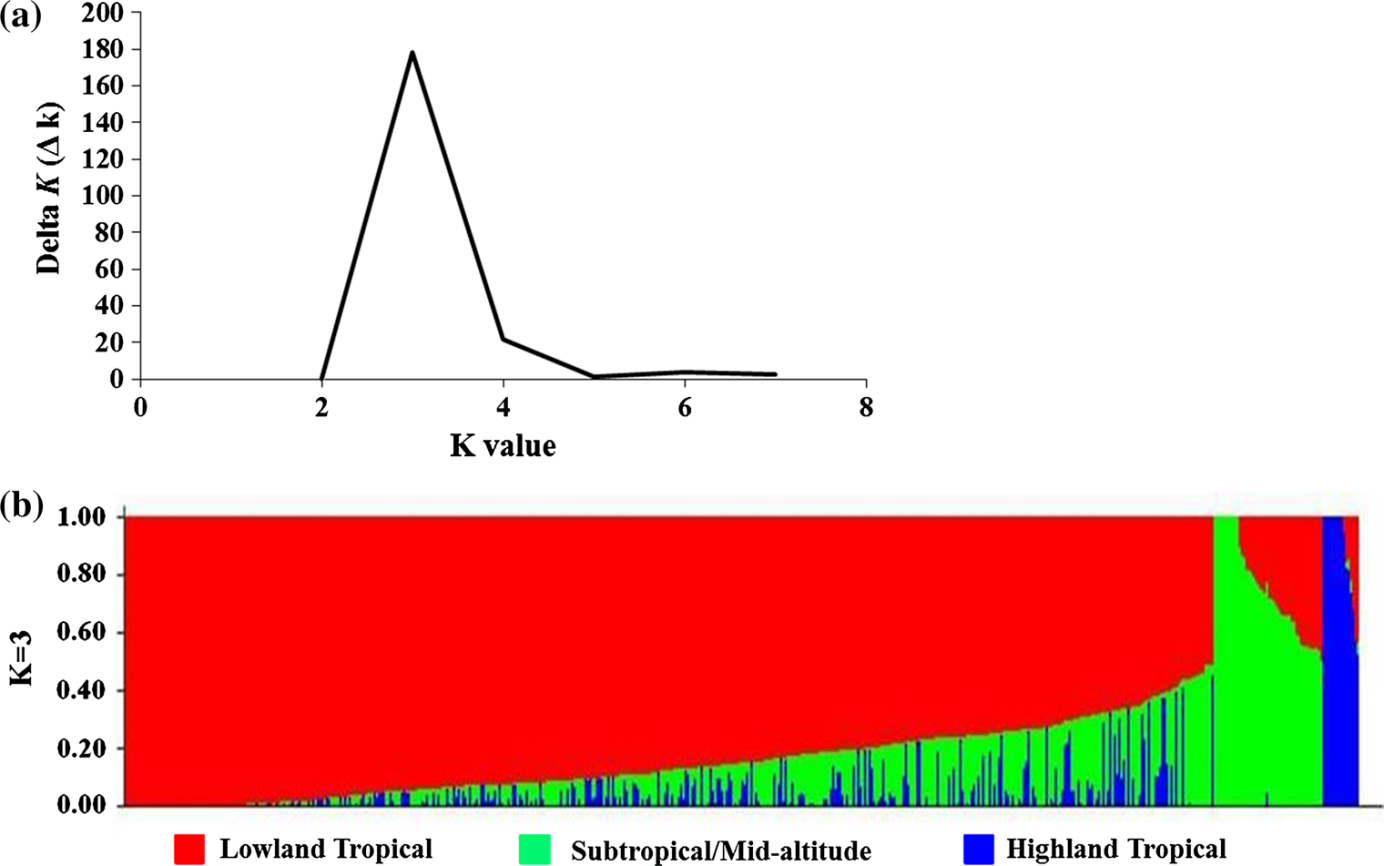
Population structure of 538 CMLs estimated with 7497 SNPs. **a** Delta *K* values for *K* ranging from 2 to 7. **b** Population structure of 538 CMLs when *K* = 3. Each of the 538 individuals is represented by a *thin vertical bar*, which is partitioned into *K* = 3 colored segments on the *x*-axis, with lengths proportional to the estimated probability membership in each of the K-inferred clusters (*y*-axis)

Results of population structure analysis of all 538 CMLs for *K* ranging from 1 to 7 are shown in Fig. 4a. The most significant peak of *ΔK* was observed when *K* = 3, which means the entire panel could be clustered into three subgroups (Lowland Tropical, Subtropical/Mid-altitude and Highland Tropical) based on environmental adaptations and CIMMYT maize breeding history. According to information of environmental adaptation and pedigree, the Lowland Tropical subgroup consists of 283 lines derived mainly from the Mexico Lowland, Asia Lowland and Africa Lowland environmental adaptations. The Subtropical/Mid-altitude subgroup consists of 225 lines derived mainly from the Mexico Subtropical, Africa Mid-altitude and South America environmental adaptations. The number of lines in the Lowland Tropical and in the Subtropical/Mid-altitude subgroups is similar to the environmental adaptation and pedigree information. However, the Lowland Tropical subgroup seems to have many more inbred lines than the Subtropical/Mid-altitude subgroup in Fig. 4b; this is because some Subtropical/Mid-altitude lines were clustered with those of the Lowland Tropical subgroup, illustrating the effect of extensive germplasm exchange that occurred between these two environmental adaptations. In fact, most of the germplasm moved from the Lowland Tropical subgroup to the Subtropical/Mid-altitude subgroup, due to reduced disease selection pressure (Fig. 4b). All 30 Highland Tropical lines are exclusively associated with the Mexico Highland Tropical environmental adaptation, indicating that germplasm exchange between the Highland Tropical adaptation and the other two environmental adaptations is rare and that the Highland Tropical adaptation is more specialized (Fig. 4b).

### Principal component analysis

**Fig. 5:**
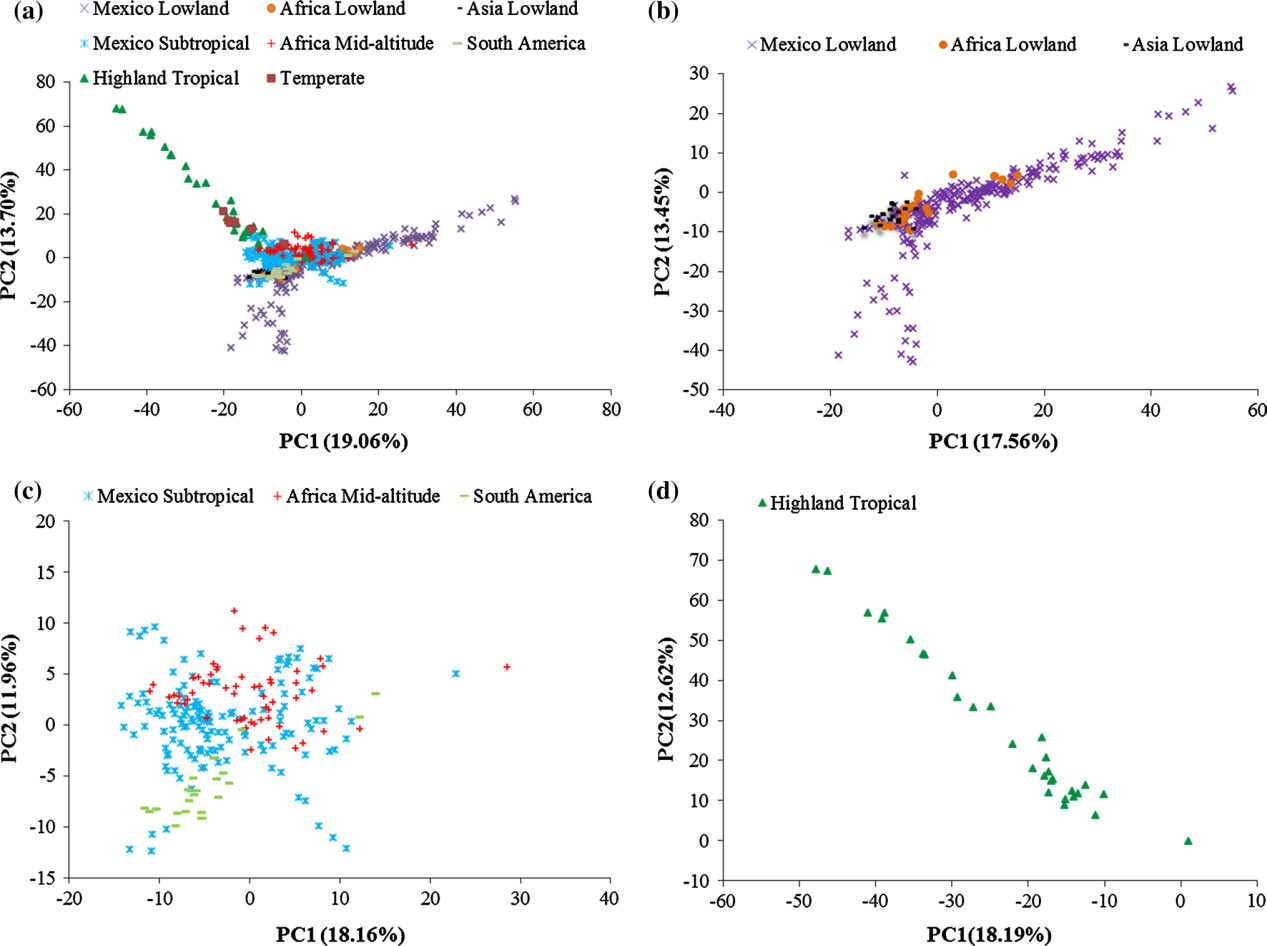
Principal component analysis (PCA) estimated with 362,008 SNPs of **a** 544 maize inbred lines consisting of 538 CMLs and 6 temperate inbred lines, **b** 283 Lowland Tropical CMLs, **c** 225 Subtropical/Mid-altitude CMLs and **d** 30 Highland Tropical CMLs

PCA results based on 362,008 SNPs clearly distinguished three major subgroups among all CMLs: Lowland Tropical, Subtropical/Mid-altitude and Highland Tropical (Fig. 5). This confirmed the population structure analysis results. The six temperate inbred lines clustered separately, but close to the Highland Tropical and Subtropical/Mid-altitude subgroups, whereas the Lowland Tropical subgroup overlapped partially with the Subtropical/Mid-altitude subgroup. The first two principal components explained 32.76, 31.01, 30.12 and 30.81 % of the total SNP variation in the entire panel, Lowland Tropical subgroup, Subtropical/Mid-altitude subgroup and Highland Tropical subgroup, respectively. The main results of further PCA analyses within each subgroup indicated that: (a) within the Lowland Tropical subgroup, the lines with Asia Lowland and Africa Lowland adaptations centrally clustered with each other; however, lines with Mexico Lowland adaptation were scattered, indicating broad genetic divergence and a trend of heterotic patterns in this adaptation; (b) within the Subtropical/Mid-altitude subgroup, lines with Mexico Subtropical and Africa Mid-altitude adaptations were scattered, revealing broad genetic divergence and a trend of heterotic patterns in these adaptations; lines CML319 and CML394 were derived from the Subtropical/Mid-altitude subgroup, but more closely overlapped with lines in the Lowland Tropical subgroup; and (c) within the Highland Tropical subgroup, most of the lines were scattered over a wide range and trended to be two clusters at the ends, which indicated a trend of heterotic patterns in this adaptation. However, this finding has to be supported by including more Highland Tropical subgroup samples into further analysis.

### Genetic distance among different subgroups

The genetic distance among different subgroups was estimated with the 362,008 SNPs (Table 4); results indicated that the genetic distance between the Tropical subgroups and Temperate subgroup is greater than the genetic distance among Tropical subgroups. The genetic distances between the Temperate subgroup and the Lowland Tropical subgroup, the Subtropical/Mid-altitude subgroup and the Highland Tropical subgroup were 0.71, 0.70 and 0.69, respectively. The greatest genetic distance was observed between the Lowland Tropical subgroup and the Temperate subgroup with a value of 0.71, and the smallest genetic distance was observed between the Lowland Tropical subgroup and the Subtropical/Mid-altitude subgroup with a value of 0.56. The genetic distance between the Highland Tropical subgroup and the Lowland Tropical subgroup was greater than the distance between the Highland Tropical subgroup and the Subtropical/Mid-altitude subgroup, which is consistent with the PCA results.

**Table 4:** Genetic distance between different subgroups

Subgroup	Lowland Tropical	Subtropical/Mid-altitude	Highland Tropical	Temperate
Lowland Tropical	0	0.56	0.63	0.71
Subtropical/Mid-altitude	–	0	0.61	0.70
Highland Tropical	–	–	0	0.69
Temperate	–	–	–	0

### Genetic characterization of each subgroup

Genetic characterization of each subgroup was quantified by calculating the average values of PIC, heterozygosity rate and gene diversity of all the lines within each subgroup (Table 3). The PICs of the three Tropical subgroups were similar, and the values were higher than that of the Temperate subgroup. In contrast to the 0 % heterozygosity rate in the Temperate subgroup, the heterozygosity rate in the three Tropical subgroups was 1 %, well within the expected ranges for residual heterozygosity found in maize inbred lines. Gene diversity of the three Tropical subgroups was similar and higher than that in the Temperate subgroup. Gene diversity values of the entire panel, the Lowland Tropical subgroup, the Subtropical/Mid-Altitude subgroup and the Highland Tropical subgroup were 0.31, 0.30, 0.30 and 0.26, respectively, whereas it was only 0.21 in the Temperate subgroup.

### Cluster analysis of all lines and important lines within each subgroup

**Fig. 6:**
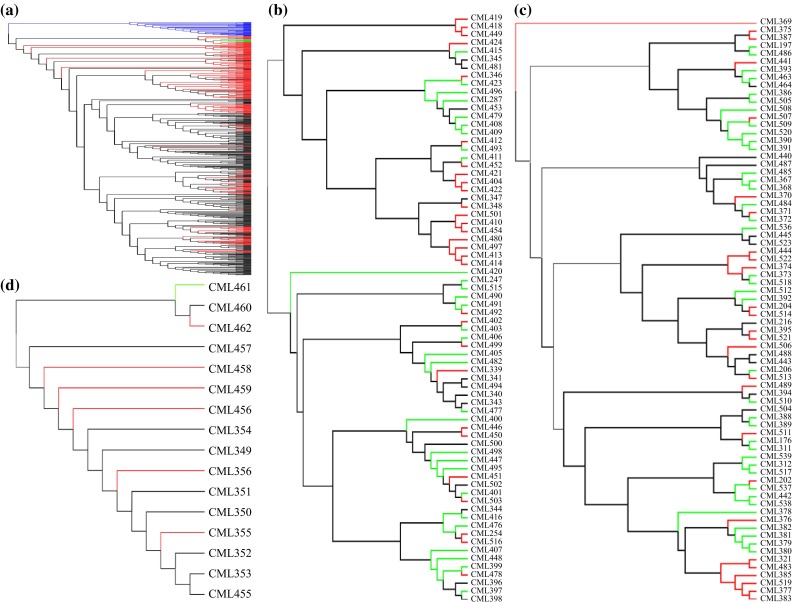
Unweighted pair group method with arithmetic mean (UPGMA) tree based on Rogers’ genetic distance calculated from 362,008 SNPs of **a** 544 maize inbred lines consisting of 538 CMLs and 6 temperate inbred lines, **b** 72 selected Lowland Tropical CMLs with high importance, **c** 74 selected Subtropical/Mid-altitude CMLs with high importance and **d** 16 selected Highland Tropical CMLs with high importance. In Fig. 6a, subgroups are indicated with different colors; Lowland Tropical lines, Subtropical/Mid-altitude lines, Highland Tropical lines and Temperate lines are represented by *black*, *red*, *blue* and *green*, respectively. In Fig. 6b, c and d, CMLs assigned to different heterotic groups are indicated with a different color. CMLs from heterotic groups A, B and AB are represented by *red*, *green* and *black*, respectively. Heterotic group information was estimated based on pedigree information and combining ability tests through diallel crosses and line-by-tester analyses

THe UPGMA tree results based on Rogers’ (1972) genetic distance for all tested lines were consistent with the main conclusions of PCA and population structure analysis (Fig. 6a). Three major groups were clearly distinguished among all the CMLs, and the Lowland Tropical subgroup overlapped partially with the Subtropical/Mid-altitude subgroup. Within each subgroup, important lines (including the most frequently used lines and testers) were selected to investigate their genetic relatedness with each other (Fig. 6b, c, d). Although the heterotic patterns of all the lines based on molecular marker cluster analysis were not fully consistent with the combining ability analysis, they showed a slight trend indicating that some lines from the same heterotic group were more closely related to each other, especially in the Lowland Tropical subgroup, where most of the lines from heterotic group A clustered at the top in Fig. 6b, while most of the lines from heterotic group B clustered at the bottom; the results of molecular marker cluster analysis were partially consistent with the heterotic group trend found in PCA.

## Discussion

GBS is a low coverage sequencing technology which results in a very high missing rate, for example, in both the 955,120 and 362,008 SNP datasets it is above 50 %, and high levels of missing data can be a problem for downstream analysis like association mapping. Generally, imputation of missing data is not necessary for genetic diversity analysis. Imputation uses information from the other haplotypes to fill the missing data gaps, so basically it uses sample relatedness to create the new dataset. Using imputed data to analyze sample relatedness could bias results, showing close haplotypes to be closer than they really are and vice versa. Additionally, more layers of error to the genotyping data happened on the SNPs toward regions of the genome with more chances of getting sequenced (i.e., duplicated regions) and also regions with more chances of getting imputed (i.e., low divergence regions) during SNP calling and imputation processes. It has been shown that even with high levels of missing rate, the GBS data generated without imputation can be used for genetic diversity and population structure analysis in the temperate maize diversity panel (Romay et al. 2013); our study firstly showed that un-imputed GBS data are also appropriate for genetic diversity analysis in the tropical maize panel.

MAF of 5 % rather than MAF of 1 % was performed in this study. There are several reasons: (1) the genotyping error of GBS in temperate maize has been reported as 0.42 % based on the data from NAM (nested association mapping) populations, where few tropical materials were included (Glaubitz et al. 2014). Genotyping error of GBS will be much increased in tropical maize, but we did not find any report about the genotyping error rate of GBS estimated in a complete tropical maize panel. In this study, the reference genome used for SNP calling is B73, which is a temperate maize inbred line. When a temperate maize inbred line is used as reference to call SNPs for tropical maize inbred lines (i.e., CML panel tested in this study), the ascertainment bias and SNP calling error of GBS increase. A higher MAF (i.e., 5 %) might be helpful for reducing ascertainment bias and selecting more reliable SNPs. (2) GBS is a low coverage sequencing technology, which caused a very high missing rate and large number of SNPs with very low frequency on the genotyped samples, especially in a broad genetic diverse panel. In this study, a higher MAF (i.e., 5 %) was performed in a broad genetic diverse panel for SNP filtering, which may reduce ascertainment bias and select more reliable SNPs for further molecular characterization work. However, a much lower MAF (i.e., 1 %) is recommended for filtering SNPs in a lower diversity panel, which may highly increase the power for further genetic diversity analysis. (3) Additionally, a very high marker density, i.e., 362,008 filtered SNPs with an average marker density of 1 SNP/6 kb, was used in this study compared with previous studies. Marker density is one of the strong points of this study on increasing the power of further analyses. Compared with MAF of 1 %, the number of filtered SNPs with MAF of 5 % decreased from 499,297 to 362,008, and higher marker density obtained by filtering SNPs with a lower MAF may not affect the overall results.

The results of the kinship analysis performed in this study showed that kinship coefficients of 64 % of the paired lines were equal to 0 and only 2 % of them were above 0.05. This information reflected the uniqueness of most inbred lines in the current CML collection, since most of the CMLs were either not related or distantly related to each other. Our kinship coefficient results are similar to those of Wen et al. (2011), who reported that about 60 % of the pairwise kinship coefficients among 359 inbred maize lines were close to zero. However, they are much lower than those of Semagn et al. (2012), who reported that 79 % of the kinship coefficients among 450 inbred maize lines ranged from 0.05 to 0.50; these authors used a maize collection with narrow genetic divergence, as all the lines were developed and released mainly by CIMMYT’s eastern and southern Africa maize breeding programs.

Previous studies have measured LD decay distance in different germplasm collections with various kinds of low-to-medium density genotyping platforms. Compared with previous studies, the average LD decay according to the physical distance in this study was more rapid, and the average LD decay distance was smaller, with an average distance of 3.76 kb in the entire panel. Yan et al. (2009) reported that the average LD decay distance was 5–10 kb in a global maize collection of 632 lines, and Wu et al. (2014) measured that the average LD decay distance was 391 kb in a collection of 367 inbred lines widely used in maize breeding of China. The LD decay distance measured in this study was much smaller than that reported in the temperate maize collection, because tropical and subtropical lines are more diverse and contain more rare alleles.

In this study, the current CML collection was molecularly characterized by performing population structure, principal component and neighbor-joining cluster analyses. The results of these analyses revealed the population structure and clear genetic divergence between temperate and tropical inbred lines, which was in agreement with previous studies (Lu et al. 2009; Wen et al. 2011,  2012). Our results also showed a clear separation by environmental adaptation. Inbred lines from the three major environmental adaptations (i.e., Lowland Tropical, Subtropical/Mid-altitude and Highland Tropical) formed clear clusters. Most lines that are related by pedigree tended to cluster into the same group, which was basically consistent with CIMMYT maize breeding history. However, our results were different from those of several previous studies, where the authors reported a lack of clear clustering patterns in the CIMMYT germplasm based on environmental adaptation or mega-environment (Semagn et al. 2012; Xia et al. 2004, 2005). These differences could be explained by our use of high-density GBS SNPs, which may have increased the resolution of the genetic characterization analysis. In this study, 362,008 filtered SNPs with an average marker density of 1 SNP/6 kb or 11 SNPs/gene were finalized and used in further genetic characterization analyses, assuming that the maize genome is about 2400 Mb and there are approximately 32,000 genes in the maize genome (Yan et al. 2009).

Gene diversity values of the three Tropical subgroups were similar and higher than those of the Temperate subgroup, and the average genetic distance between the Temperate subgroup and each of the Tropical subgroups was greater than that between the Tropical subgroups. The greatest genetic distance was observed between the Lowland Tropical and Temperate subgroups, and the smallest genetic distance was observed between the Lowland Tropical and Subtropical/Mid-altitude subgroups. These observations are consistent with the current germplasm exchange patterns where there is constant flow of germplasm from the tropical program into the subtropical with little to no exchange with the highland program. The genetic distance between the Temperate subgroup and the Highland Tropical subgroup was smaller than that between the Temperate subgroup and the other two subgroups. Germplasm rarely occurs between the Temperate breeding program and highland tropical breeding program, because the Highland Tropical subgroup has narrow adaptation and the genetic distances estimated between the Temperate subgroup with other subgroups are not very accurate; especially with the Highland subgroup, small sample size of these two subgroups affected the accuracy of genetic distance estimation. In practice, Temperate breeding programs more frequently exchange germplasm with the Subtropical/Mid-altitude breeding program than the other breeding programs. These analyses revealed ample natural genetic diversity in tropical maize germplasm, suggesting that the current CML collection could be an important resource to help drive future genetic gains in maize breeding programs worldwide.

It has been shown that SSRs provide higher resolution in genetic diversity analyses, given that the power of one SSR is similar to that of ten SNPs for estimating population structure and relative kinship (Lu et al. 2009; Yan et al. 2009). This is because SNPs from array based on allele sharing are lower than the more polymorphic SSRs, and the maximum number of alleles per locus is restricted to two for bi-allelic SNPs. However, a number of SNP alleles may perform better than the same number of SSR alleles, since many more SNP loci with two alleles provide better genome coverage than a lower number of SSR loci with more alleles per locus. More weight should thus be given in genetic diversity analysis to the number of loci than to the number of alleles (Lu et al. 2009). Larger numbers of SNPs are required to replace the highly polymorphic SSRs. Several previous studies have shown the efficiency and power of SNP markers in genetic diversity analyses (Lu et al. 2009, 2013; Romay et al. 2013; Semagn et al. 2012; Wu et al. 2014). However, the use of SNP genotyping chips may cause ascertainment bias, which means markers developed to be polymorphic in one set of germplasm are likely to provide a biased estimate of diversity in another set of germplasm (Lu et al. 2009). The only way to fully remove the bias is to do de novo sequencing on all the samples; however, the cost of this is still prohibitive. Next-generation sequencing technologies, such as GBS, make low-cost, low-coverage, whole-genome sequencing widely available, which could reduce to some extent ascertainment bias in maize molecular characterization studies. Romay et al. (2013) genotyped 2815 maize inbred accessions from the USA national maize inbred seed bank with GBS, and 681,257 SNPs were developed and successfully used for analyzing the genetic diversity and population structure of this publicly available maize collection and for performing genome-wide association studies on simple and complex inherited traits. In this study, a large and diverse collection of 539 CMLs were genotyped with 955,690 GBS SNPs; these SNPs were called using haplotype information from a collection of more than 60,000 maize samples (the AllZeaGBSv2.7 Production Build) including temperate and tropical germplasm. The reference genome is from B73, a temperate maize inbred line. Of the 955,690 SNPs, 62 % in the collection are rare, which is a litter higher than the number found by Romay et al. (2013), who reported that more than half the SNPs are rare.

In the current collection, only about half the CMLs (i.e., 243 of 538) have heterotic information estimated based on pedigree information and combining ability tests through diallel and line-by-tester analyses. Since only a limited number of lines can be included in each combining ability test experiment, it is not possible to estimate the heterotic group and genetic relatedness of all maize lines in the current CML collection via one general combining ability test. Molecular marker analyses provide an alternative approach for large-scale genetic diversity characterization within a given germplasm collection. However, it has been reported several times that the heterotic patterns in tropical maize collections are still not clear compared with temperate maize, and the heterotic patterns estimated based on molecular markers are not fully consistent with those estimated based on combining ability tests and pedigree information (Lu et al. 2009; Semagn et al. 2012; Wen et al. 2012). In this study, we measured the genetic relatedness among all CMLs and our results confirmed this conclusion: the GBS SNPs were unable to separate heterotic groups A and B that were established based on combining ability tests. The difficulties of assigning lines to different heterotic groups are due to the diverse original and incomplete pedigree information, regardless of the marker system used. Shorter hybrid-oriented breeding history for tropical germplasm and use of different testers across breeding programs are probably the other important reasons. The same line can be heterotic group A or B depending on the tester used, which may result in mixing up of heterotic groups.

The creation of heterotic groups in maize is based on long-term selection. The development of heterotic groups in temperate maize started around 100 years ago, but heterotic group development work in tropical maize at CIMMYT began only three decades ago, in the mid-1980s. Most CMLs were derived from broad germplasm pools, populations and open-pollinated varieties; only more recently released CMLs were developed from bi-parental crosses using the pedigree breeding method. So it is easy to understand why heterotic patterns in tropical maize are still not clear. But we also found that most of the lines from heterotic group A and heterotic group B tend to cluster together in the Lowland Tropical and Subtropical/Mid-altitude subgroups, respectively. This suggests that short-term selection for hybrid performance has contributed to classifying tropical maize heterotic patterns at CIMMYT. Therefore, combining the current heterotic information based on combining ability tests and the genetic relationships inferred from molecular marker analyses may be the best strategy to define heterotic groups for future tropical maize improvement. The results of this research will also help breeders to understand how to utilize all the CMLs in other breeding activities, such as selecting parental lines, replacing appropriate testers, assigning heterotic groups and creating a core set of germplasm.
